# Early Detection and Classification of Tomato Leaf Disease Using High-Performance Deep Neural Network

**DOI:** 10.3390/s21237987

**Published:** 2021-11-30

**Authors:** Naresh K. Trivedi, Vinay Gautam, Abhineet Anand, Hani Moaiteq Aljahdali, Santos Gracia Villar, Divya Anand, Nitin Goyal, Seifedine Kadry

**Affiliations:** 1 Chitkara University Institute of Engineering and Technology, Chitkara University, Rajpura 140401, India; nareshk.trivedi@chitkara.edu.in (N.K.T.); abhineet.anand@chitkara.edu.in (A.A.); 2 School of Computing, DIT University, Dehradun 248009, India; vinay.gautam@dituniversity.edu.in; 3 Faculty of Computing and Information Technology, King Abdulaziz University, Jeddah 37848, Saudi Arabia; Hmaljahdali@kau.edu.sa; 4 Higher Polytechnic School/Industrial Organization Engineering, Universidad Europea del Atlántico, Isabel Torres 21, 39011 Santander, Spain; santos.gracia@uneatlantico.es; 5 Department of Project Management, Universidad Internacional Iberoamericana, Campeche 24560, Mexico; 6 Department of Computer Science and Engineering, Lovely Professional University, Phagwara 144411, India; divyaanand.y@gmail.com; 7 Faculty of Applied Computing and Technology, Noroff University College, 4608 Kristiansand, Norway; seifedine.kadry@noroff.no

**Keywords:** image processing, convolution neural network, plant leaf disease, deep learning, artificial intelligence

## Abstract

Tomato is one of the most essential and consumable crops in the world. Tomatoes differ in quantity depending on how they are fertilized. Leaf disease is the primary factor impacting the amount and quality of crop yield. As a result, it is critical to diagnose and classify these disorders appropriately. Different kinds of diseases influence the production of tomatoes. Earlier identification of these diseases would reduce the disease’s effect on tomato plants and enhance good crop yield. Different innovative ways of identifying and classifying certain diseases have been used extensively. The motive of work is to support farmers in identifying early-stage diseases accurately and informing them about these diseases. The Convolutional Neural Network (CNN) is used to effectively define and classify tomato diseases. Google Colab is used to conduct the complete experiment with a dataset containing 3000 images of tomato leaves affected by nine different diseases and a healthy leaf. The complete process is described: Firstly, the input images are preprocessed, and the targeted area of images are segmented from the original images. Secondly, the images are further processed with varying hyper-parameters of the CNN model. Finally, CNN extracts other characteristics from pictures like colors, texture, and edges, etc. The findings demonstrate that the proposed model predictions are 98.49% accurate.

## 1. Introduction

Plants are an integral part of our lives because they produce food and shield us from dangerous radiation. Without plants, no life is imaginable; they sustain all terrestrial life and defend the ozone layer, which filters ultraviolet radiations. Tomato is a food-rich plant, a consumable vegetable widely cultivated [1]. Worldwide, there are approximately 160 million tons of tomatoes consumed annually [2]. The tomato, a significant contributor to reducing poverty, is seen as an income source for farm households [3]. Tomatoes are one of the most nutrient-dense crops on the planet, and their cultivation and production have a significant impact on the agricultural economy. Not only is the tomato nutrient-dense, but it also possesses pharmacological properties that protect against diseases such as hypertension, hepatitis, and gingival bleeding [1]. Tomato demand is also increasing as a result of its widespread use. According to statistics, small farmers produce more than 80% of agricultural output [2]; due to diseases and pests, about 50% of their crops are lost. The diseases and parasitic insects are the key factors impacting tomato growth, making it necessary to research the field crop disease diagnosis.

The manual identification of pests and pathogens is inefficient and expensive. Therefore, it is necessary to provide automated AI image-based solutions to farmers. Images are being used and accepted as a reliable means of identifying disease in image-based computer vision applications due to the availability of appropriate software packages or tools. They process images using image processing, an intelligent image identification technology which increases image recognition efficiency, lowers costs, and improves recognition accuracy [3].

Although plants are necessary for existence, they experience numerous obstacles. An early and accurate diagnosis helps decrease the risk of ecological damage. Without systematic disease identification, product quality and quantity suffer. This has a further detrimental effect on a country’s economy [1]. Agricultural production must expand by 70% by 2050 to meet global food demands, according to the United Nations Food and Agriculture Organization (FAO) [2]. In opposition, chemicals used to prevent diseases, such as fungicides and bactericides, negatively impact the agricultural ecosystem. We therefore need quick and effective disease classification and detection techniques that can help the agro-ecosystem. Advance disease detection technology, such as image processing and neural networks, will allow the design of systems capable of early disease detection for tomato plants. The plant production can be reduced by 50% due to stress as a result [1]. Inspecting the plant is the first step in finding disease, then figuring out what to work with based on prior experience is the next step [3]. This method lacks scientific consistency because farmers’ backgrounds differ, resulting in the process being less reliable. There is a possibility that farmers will misclassify a disease, and an incorrect treatment will damage the plant. Similarly, field visits by domain specialists are pricey. There is a need for the development of automated disease detection and classification methods based on images that can take the role of the domain expert.

It is necessary to tackle the leaf disease issue with an appropriate solution [4,5]. Tomato disease control is a complex process that takes constant account of a substantial fraction of production cost during the season [6,7,8,9]. Vegetable diseases (bacteria, late mildew, leaf spot, tomato mosaic, and yellow curved) are prevalent. They seriously affect plant growth, which leads to reduced product quality and quantity [10]. As per past research, 80–90% of diseases of plants appear on leaves [11]. Tracking the farm and recognizing different forms of the disease with infected plants takes a long time. Farmers’ evaluation of the type of plant disease might be wrong. This decision could lead to insufficient and counterproductive defense measures implemented in the plant. Early detection can reduce processing costs, reduce the environmental impact of chemical inputs, and minimize loss risk [12,13,14].

Many solutions have been proposed with the advent of technology. Here in this paper, the same solutions are used to recognize leaf diseases. Compared with other image regions, the main objective is to make the lesion more apparent. Problems such as (1) shifts in illumination and spectral reflectance, (2) poor input image contrast, and (3) image size and form range have been encountered. Pre-processing operations include image contrast, greyscale conversion, image resizing, and image cropping and filtering [15,16,17]. The next step is the division of an image into objects. These objects are used to determine regions of interest as infected regions in the image [18]. Unfortunately, the segmentation method has many problems:

- When the conditions of light differ from eligible photographs, color segmentation fails.
- Regional segmentation occurs because of initial seed selection.
- Texture varieties take too long to handle.

The next step for classification is to determine which class belongs to the sample. Then, one or more different input variables of the procedure are surveyed. Occasionally, the method is employed to identify a particular type of input. Improving the accuracy of the classification is by far the most extreme classification challenge. Finally, the actual data are used to create and validate datasets dissimilar to the training set.

The rest of the paper is organized as follows: Section 2 reviews the extant literature. Then, the material method and process are described in Section 3. Next, the results analysis and discussion are explained in Section 4. Finally, Section 5 is the conclusion.

## 2. Related Work

Various researchers have used cutting-edge technology such as machine learning and neural network architectures like Inception V3 net, VGG 16 net, and SqueezeNet to construct automated disease detection systems. These use highly accurate methods for identifying plant disease in tomato leaves. In addition, researchers have proposed many deep learning-based solutions in disease detection and classification, as discussed below in [19,20,21,22,23,24,25,26,27,28,29,30,31,32,33,34,35,36,37,38,39,40,41,42,43,44,45,46,47,48].

A pre-trained network model for detecting and classifying tomato disease has been proposed with 94–95% accuracy [19,20]. The Tree Classification Model and Segmentation is used to detect and classify six different types of tomato leaf disease with a dataset of 300 images [21]. A technique has been proposed to detect and classify plant leaf disease with an accuracy of 93.75% [22]. The image processing technology and classification algorithm detect and classify plant leaf disease with better quality [23]. Here, an 8-mega-pixel smartphone camera is used to collect sample data and divides it into 50% healthy and 50% unhealthy categories. The image processing procedure includes three elements: improving contrast, segmenting, and extracting features. Classification processes are performed via an artificial neural network using a multi-layer feed-forward neural network, and two types of network structures are compared. The result was better than the Multilayer Perceptron (MLP) network and Radial Basis Function (RBF) network results. The quest divides the plant blade’s picture into healthy and unhealthy; it cannot detect the form of the disease. Authors used leaf diseases to identify and achieve 87.2% classification accuracy through color space analysis, color time, histogram, and color coherence [24].

AlexNet and VGG 19 models have been used to diagnose diseases affecting tomato crops using a frame size of 13,262. The model is used to achieve 97.49% precision [25]. A transfer learning and CNN Model is used to accurately detect diseases infecting dairy crops, reaching 95% [26]. A neural network to determine and classify tomato plant leaf conditions using transfer learning as an AlexNet based deep learning mechanism achieved an accuracy of 95.75% [27,28]. Resnet-50 was designed to identify 1000 diseases of tomato leaf, i.e., a total of 3000 pictures with the name of lesion blight, late blight, and the yellow curl leaf. The Network Activation function for comparison has been amended to Leaky-ReLU, and the kernel size has been updated to 11 × 11 for the first convolution layer. The model predicts the class of diseases with an accuracy of 98.30% and a precision of 98.0% after several repetitions [29]. A simplified eight-layered CNN model has been proposed to detect and classify tomato leaf disease [30]. In this paper, the author utilized the PlantVillage dataset [31] that contains different crop datasets. The tomato leaf dataset was selected and used to performe deep learning; the author used the disease classes and achieved a better accuracy rate.

A simple CNN model with eight hidden layers has been used to identify the conditions of a tomato plant. The proposed techniques show optimal results compared to other classical models [32,33,34,35]. The image processing technique uses deep learning methods to identify and classify tomato plant diseases [36]. Here, the author used the segmentation technique and CNN to implement a complete system. A variation in the CNN model has been adopted and applied to achieve better accuracy.

LeNet has been used to identify and classify tomato diseases with minimal resource utilization in CPU processing capability. Furthermore, the automatic feature extraction technique has been employed to improve classification accuracy [37]. ResNet 50 model has been used to classify and identify tomato disease. The authors detected the diseases in multiple steps: Firstly, by segregating the disease dataset. Secondly, by adapting and adjusting the model based on the transfer learning approach, and lastly, by enhancing the quality of the model by using data augmentation. Finally, the model is authenticated by using the dataset. The model outperformed various legacy methods and achieved 97% accuracy [38]. Hyperspectral images identify rice leaf diseases by evaluating different spectral responses of leaf blade fractions and identifying Sheath blight (ShB) leaf diseases [39]. A spectral library has been created using different disease samples [40]. An improved VGG16 has been used to identify apple leaf disease with an accuracy rate of 99.01% [41].

The author employed image processing, segmentation, and a CNN to classify leaf disease. This research attempts to identify and classify tomato diseases in fields and greenhouse plants. The author used deep learning and a robot in real-time to identify plant diseases utilizing the sensor’s image. AlexNet and SqueezeNet are deep learning architectures used to diagnose and categorize plant disease [42]. The authors built convolutional neural network models using leaf pictures of healthy and sick plants. An open-source PlavtVillage dataset with 87,848 images of 25 plants classified into 58 categories and a model was used to identify plant/disease pairs with a 99.53% success rate (or healthy plant). The authors suggest constructing a real-time plant disease diagnosis system based on the proposed model [43].

In this paper, the authors reviewed all CNN variants for plant disease classification. The authors also briefed all deep learning principles used for leaf disease identification and classification. The authors mainly focused on the latest CNN models and evaluated their performance. Here, the authors summarized CNN variants such as VGG16, VGG19, and ResNet. In this paper, the authors discuss pros, cons, and future aspects of different CNN variants [44].

This work is mainly focused on investigating an optimal solution for plant leaf disease detection. This paper proposes a segmentation-based CNN to provide the best solution to the defined problem. This paper uses segmented images to train the model compared to other models trained on the complete image. The model outperformed and achieved 98.6% classification accuracy. The model was trained and tested on independent data with ten disease classes [45].

A detailed learning technique for the identification of disease in tomato leaves using enhanced CNNs is presented in this article.

1. The dataset for tomato leaves is built using data augmentation and image annotation tools. It consists of laboratory photos and detailed images captured in actual field situations.
2. The recognition of tomato leaves is proposed using a Deep Convolutional Neural Network (DCNN). Rainbow concatenation and GoogLeNet Inception V3 structure are all included.
3. In the proposed INAR-SSD model, the Inception V3 module and Rainbow concatenation detect these five frequent tomato leaf diseases.

The testing results show that the INAR-SSD model achieves a detection rate of 23.13 frames per second and detection performance of 78.80% mAP on the Apple Leaf Disease Dataset (ALDD). Furthermore, the results indicate that the innovative INAR-SSD (SSD with Inception module and Rainbow concatenation) model produces more accurate and faster results for the early identification of tomato leaf diseases than other methods [46].

An EfficientNet, a convolutional neural network with 18,161 plain segmented tomato leaf images, is used to classify tomato diseases. Two leaf segmentation models, U-net and Modified U-net, are evaluated. The models’ ability was examined categorically (healthy vs disease leaves and 6- and 10-class healthy vs sick leaves). The improved U-net segmentation model correctly classified 98.66% of leaf pictures for segmentation. EfficientNet-B7 surpassed 99.95% and 99.12% accuracy for binary and six-class classification, and EfficientNet-B4 classified images for ten classes with 99.89 percent accuracy [47].

Disease detection is crucial for crop output. Therefore, disease detection has led academics to focus on agricultural ailments. This research presents a deep convolutional neural network and an attention mechanism for analyzing tomato leaf diseases. The network structure has attention extraction blocks and modules. As a result, it can detect a broad spectrum of diseases. The model also forecasts 99.24% accuracy in tests, network complexities, and real-time adaptability [48].

Convolutional Neural Networks (CNNs) have revolutionized image processing, especially deep learning methods. Over the last two years, numerous potential autonomous crop disease detection applications have emerged. These models can be used to develop an expert consultation app or a screening app. These tools may help enhance sustainable farming practices and food security. The authors looked at 19 studies that employed CNNs to identify plant diseases and assess their overall utility [49].

To depict the illustrations, the authors depended on the PlantVillage dataset. The authors did not evaluate the performance of the neural network topologies using typical performance metrics such as F1-score, recall, precision, etc. Instead, they assessed the model’s accuracy and inference time. This article proposes a new deep neural network model and evaluates it using a variety of evaluation metrics.

## 3. Materials and Methods

In this part, cutting-edge methodologies, models, and datasets are utilized to attain outcomes.

### 3.1. Dataset

**Figure 1:**
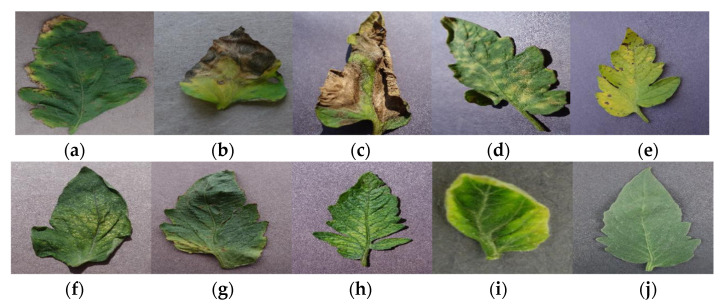
Sample leaf image with disease and pathogen for (**a**) Bacterial_Spot(Xanthomonas vesicatoria), (**b**) Early_Blight(fungus Alternaria solani), (**c**) Late_Blight(*Phytophthora infestans*), (**d**) Leaf_Mold(Cladosporium fulvum), (**e**) Septoria_Leaf_Spot(fungus Septoria lycopersici), (**f**) Spider_Mites(floridana), (**g**) Target_Spot(fungus Corynespora), (**h**) Tomato_Mosaic_Virus(Tobamovirus), (**i**) Tomato_Yellow_Leaf_Curl_Virus(genus Begomovirus), (**j**) Healthy_Leaf.

There were ten unique classes of disease in the sample. Tomato leaves of nine types were infected, and one class was resistant. We used reference photos and disease names to identify our dataset classes, as shown in Figure 1.

In the experiment, the complete dataset was divided in the ratio of 80:20 for training and testing and validation data.

### 3.2. Image Pre-Processing and Labelling

Before training the model, image pre-processing was used to change or boost the raw images that needed to be processed by the CNN classifier. Building a successful model requires analyzing both the design of the network and the format of input data. We pre-processed our dataset so that the proposed model could take the appropriate features out of the image. The first step was to normalize the size of the picture and resize it to 256 × 256 pixels. The images were then transformed into grey. This stage of pre-processing means that a considerable amount of training data are required for the explicit learning of the training data features. The next step was to group tomato leaf pictures by type, then mark all images with the correct acronym for the disease. In this case, the dataset showed ten classes in test collection and training.

### 3.3. Training Dataset

**Figure 2:**
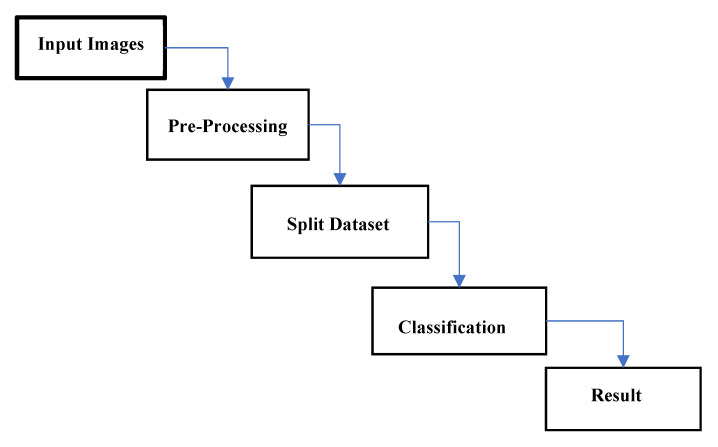
Classifier model used.

Preparing the dataset was the first stage in processing the existing dataset. The Convolutional Neural Network process was used during this step as image data input, which eventually formed a model that assessed performance. Normalization steps on tomato leaf images are shown in Figure 2.

### 3.4. Convolutional Neural Network

**Figure 3:**
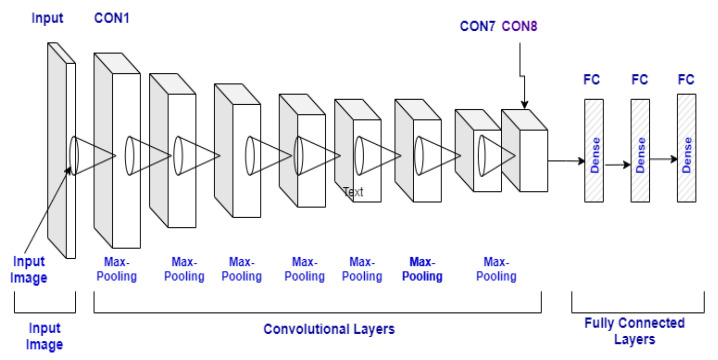
CNN model architecture.

The CNN is a neural network technology widely employed today to process or train the data in images. The matrix format of the Convolution is designed to filter the pictures. For data training, each layer is utilized in the Convolution Neural Network, including the following layers: input layer, convo layer, fully connected layer pooling layer, drop-out layer to build CNN, and ultimately linked dataset classification layer. It can map a series of calculations to the input test set in each layer. The complete architecture is shown in Figure 3, and a description of the model is in Table 1.

#### 3.4.1. Convolutional Layer

A convolution layer is used to map characteristics using the convolution procedure with the presentation layer. Each function of the map is combined with several input characteristics. Convolution can be defined as a two-function operation and constitutes the basis of CNNs. Each filter is converted to each part of the input information, and a map or 2D function map is generated. The complexity of the model encounters significant layer convolutional performance optimization. Calculated in the following equation for input z of the *i*th coalescent layer (1):

(1) $$m_{i}=\;f(Q{\;}_{i}\times z)\times z$$

Where × is a convolution operation and *f* is used for an activation function, and ***Q*** is a layer kernel convolution. $w_{i}=\left[Q_{i1},\;Q_{i2},\;\ldots ,\;Q_{iJ}\right]$, J  is the kernel layer convolution amount. Each kernel of ***Q**_i_* is a weight matrix ***K × K × L***. The number of input channels is *K* as the window size.

#### 3.4.2. Pooling Layer

The pooling layer increases the number of parameters exponentially to maximize and improve precision. Furthermore, with growing parameters, the size of the maps is reduced. The pooling layer reduces the overall output of the convolution layer. It reduces the number of training parameters by considering the spatial properties of an area representing a whole country. It also distributes the total value of all R activations to the subsequent activation in the chain. In the m-th max-pooled band, there are J-related filters that are combined.

(2) $$p_{m}=\left[p_{1,m},p_{2,m},\;\ldots ,\;p_{j,m}\right]\;\in \;R^{j}\;$$

(3) $$p_{j,m}=\max\left(h_{j(m-1)N}+r\right)$$

where N ∈ {1, …., R} is pooling shift allowing for overlap between pooling zones where N < R. It reduces the output dimensionality from K convolution bands to ***M*** = ((***K*** - ***R***))/(***N*** + 1) pooled bands and the resulting layer is ***p*** = [***p***_1, …, ***p**_**m***] ∈ ***R***^(***M***.***J***.)

Finally, a maximum of four quadrants indicates the value maximum with average pooling results.

#### 3.4.3. Fully Connected Layer

Each layer in the completely connected network is connected with its previous and subsequent layers. The first layer of the utterly corresponding layer is connected to each node in the pooling layer’s last frame. The parameters used in the CNN model take more time because of the complex computation; it is the critical drawback of the fully linked sheet. Thus, the elimination of the number of nodes and links will overcome these limitations. The dropout technique will satisfy deleted nodes and connections.

#### 3.4.4. Dropout

An absence is an approach in which a randomly selected neuron is ignored during training, and they are “dropped out” spontaneously. This means that they are briefly omitted from their contribution to the activation of the downstream neurons on the forward transfer, and no weight changes at the back are applied to the neuron. Thus, it avoids overfitting and speeds up the process of learning. Overfitting is when most data has achieved an excellent percentage through the training process, but a difference in the prediction process occurs. Dropout occurs when a neuron is located in the network in the hidden and visible layers.

**Performance Evaluation Metrics**. The accuracy, precision, recall, and F1-score measures are used to evaluate the model’s performance. To avoid being misled by the confusion matrix, we applied the abovementioned evaluation criteria.

- ***Accuracy***. Accuracy (*A_cc_*) is a measure of the proportion of accurately classified predictions that have been made so far. It is calculated as follows:

$$Acc=\frac{TP+TN}{TP+TN+FP+FN}.\;$$

Note that abbreviations such as “*TP*”, “*TN*”, “*FP*”, and “*FN*” stand for “true positive”, “true negative”, “false positive”, and “false negative”, respectively.

- ***Precision***. Precision (*Pre*) is a metric that indicates the proportion of true positive outcomes. It is calculated as follows:

$$Pre=\frac{TP}{TP+FN}\;$$

- ***Recall***. Recall (*Re*) is a metric that indicates the proportion of true positives that were successfully detected. It is calculated as follows:

$$Re=\frac{TP}{TP+FN}.\;$$

- ***F1-Score.*** The *F1-Score* is calculated as the harmonic mean of precision and recall and is defined as follows:

(4) $$F1–Score=\frac{Pre.Re}{Pre+Re}\;$$

**Proposed Algorithm:** Steps involved for Disease Detection

**Step 1**: Input color of the image *I_RGB_* of the leaf procure from the PlantVillage dataset.
**Step 2**: Given *I_RGB_*, generate the mask *M_veq_* using CNN-based segmentation.
**Step 3**: Encrust *I_RGB_* with *M_veq_* to get *M_mask_*.
**Step 4**: Divide the image *M_mask_* into smaller regions *K_tiles_* (square tiles).
**Step 5:** Classify *K_tiles_* from *M_mask_* into Tomato.
**Step 6**: Finally, *K_tiles_* is the leaf part to detect disease.
**Step 7**: Stop.

The disease detection starts with inputted image *I_RGB_* from the multiclass dataset. After input image *I_RGB_* is the mask segmented *M_veq_* using CNN. The mask image is divided into a different region *K_tiles_*. Afterward, it selects the Region of Interest (RoI), and the same is used to detect leaf disease.

The proposed algorithm for disease detection is given below:**Algorithm:** Disease Detection **Input:**$\;\mathrm{Take}\;\mathrm{different}\;\mathrm{classes}\;\mathrm{of}\;\mathrm{color}\;\mathrm{images}\;\mathrm{with}\;\mathrm{disease}\;I_{RGB\;}$ acquired from a dataset**Output:** Disease detection; (a)$\mathrm{For}\;\mathrm{given}\;\mathrm{input}\;\mathrm{image}\;(I_{RGB\;}$$),\;\mathrm{generate}\;\mathrm{the}\;\mathrm{masking}\;(M_{veg}$) using CNN-based seg mentation;
(b)$\mathrm{Overlay}\;I_{RGB\;}$$\;\mathrm{with}\;M_{veg}$$\;\mathrm{to}\;\mathrm{get}\;M_{mask}$;(c)$\mathrm{Divide}\;\mathrm{the}\;\mathrm{image}\;M_{mask}$$\;\mathrm{into}\;\mathrm{smaller}\;\mathrm{regions}\;K_{tiles}$ (square tiles);(d)$\mathrm{for}\;(K_{tiles}\;$$\mathrm{in}\;M_{mask}$)$\mathrm{Classify}\;K_{tiles}\;$$\mathrm{into}\;M_{mask}$ tomato diseases;(e)$\mathrm{if}\;K_{tiles}\;$is a disease, identify disease;(f)end.

## 4. Results Analysis and Discussion

The complete experiment was performed on Google Colab. The result of the proposed method is described with different test epochs and learning rates and explained in the next sub-section.

**Figure 4:**
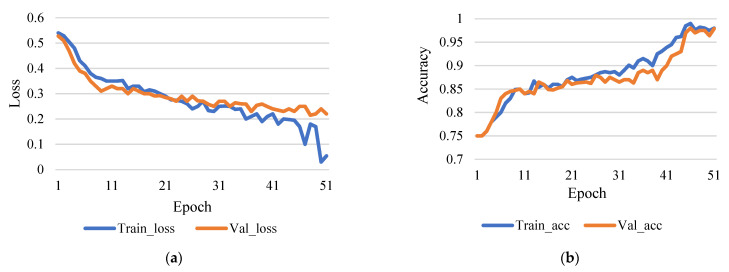
(**a**) Training loss vs validation loss (rate of learning 0.0001 and epoch 50). (**b**) Training accuracy vs validation accuracy (rate of learning 0.0001 and epoch 50).

This research used epoch 50 and epoch 100 for comparison, though learning rates were 0.0001. Figure 4a shows the comparison between training and validation loss, and Figure 4b shows the comparison between training accuracy and validation accuracy.

**Figure 5:**
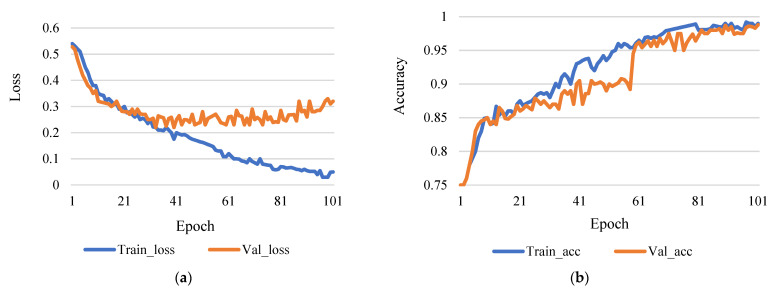
(**a**) Training loss vs validation loss (rate of learning 0.0001 and epoch 100). (**b**) Training accuracy vs validation accuracy (rate of learning 0.0001 and epoch 100).

Figure 5a shows the comparison between training loss and validation loss, and Figure 5b shows training accuracy and validation accuracy. Here, Figure 5b shows that the accuracy rate of 98.43% is achieved with a training step at 100 epochs and the rate of learning 0.0001. Therefore, it is reasonable to infer that more iterations will result in higher data accuracy based on the research technique. However, the number of epochs increases as the training phase lengthens.

**Figure 6:**
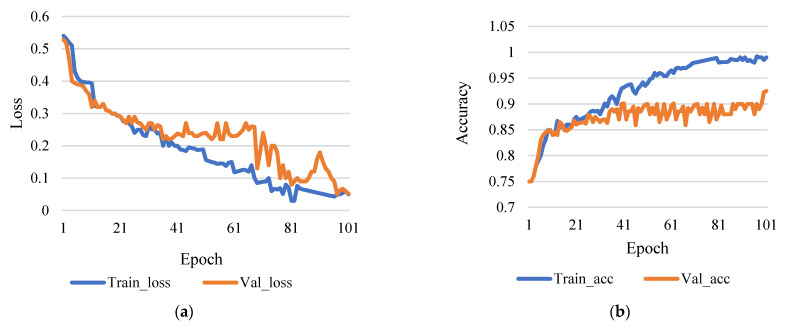
(**a**) Training loss vs validation loss (rate of learning 0.001 and epoch 50). (**b**) Training accuracy vs validation accuracy (rate of learning 0.001 and epoch 50).

This assessment looks to evaluate how machine learning plays a role in the process. For example, one of the training variables used to calculate the weight correction value for the course is the learning rate (1). This test is based on the epochs 50 and 100, while the learning rates are 0.001 and 0.01 used for comparison. Figure 6a shows the comparison between training loss and validation loss, and Figure 6b shows training accuracy and validation accuracy.

**Figure 7:**
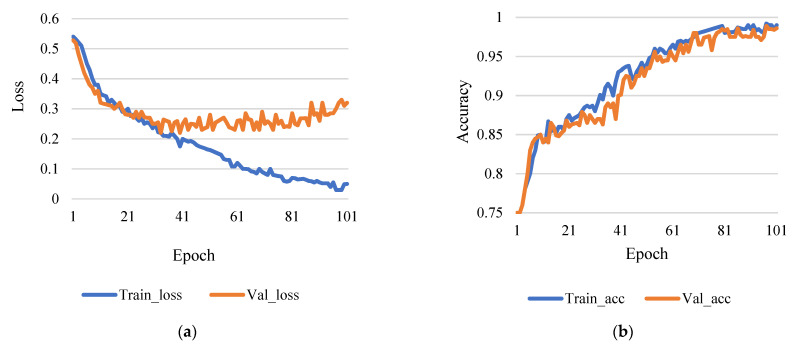
(**a**) Training loss vs validation loss (rate of learning 0.001 and epoch 100). (**b**) Training accuracy vs validation accuracy (rate of learning 0.001 and epoch 100).

**Figure 8:**
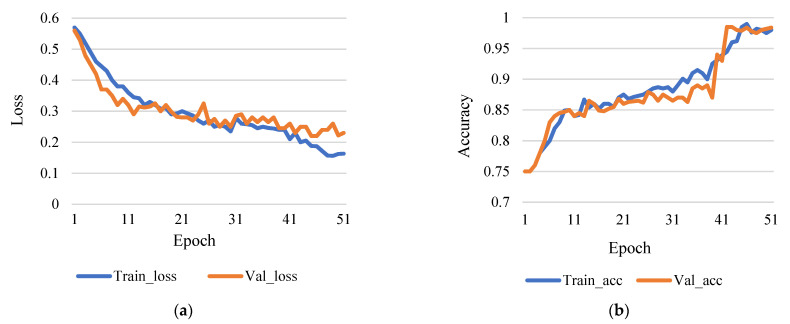
(**a**) Training loss vs validation loss (rate of learning 0.01 and epoch 50). (**b**) Training accuracy vs validation accuracy (rate of learning 0.01 and epoch 50).

**Figure 9:**
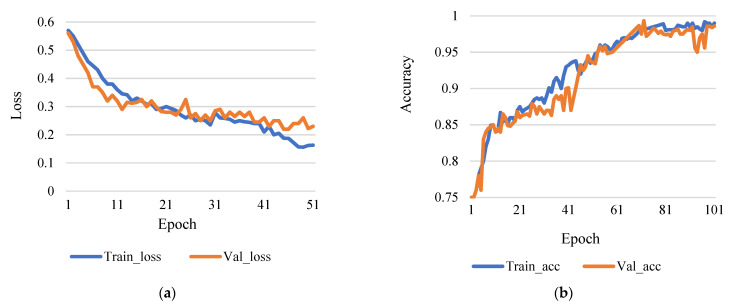
(**a**) Training loss vs validation loss (rate of learning 0.01 and epoch 100). (**b**) Training accuracy vs validation accuracy (rate of learning 0.01 and epoch 100).

Figure 7a shows the comparison between training loss and validation loss, and Figure 7b shows training accuracy and validation accuracy. According to Figure 7b, the accurate rate of 98.42% is indicated by step 50 and a learning rate of 0.001. Furthermore, Figure 8a shows the comparison between training loss and validation loss, and Figure 8b shows training accuracy and validation accuracy. Figure 8b shows that a level of accuracy of 98.52% is achieved. Figure 9a shows the training and validation losses, and Figure 9b shows training and validation accuracy. It also shows an accuracy rate of 98.5% with the 100 steps and 0.01 learning rate. Based on the assessment process used, it can evaluate a more accurate percentage of data with a greater learning rate.

As shown in the results of Table 2, the accuracy rate is dependent on both the learning rate and the epoch: The more significant the epoch value, the more precise the calculation. Table 2 describes that the experiment was performed by varying different parameters such as epoch (two values) and learning rate (three values). Different learning rates were used to find out detection accuracy. The accuracy of the experiment on different variations values is shown in Table 2.

**Figure 10:**
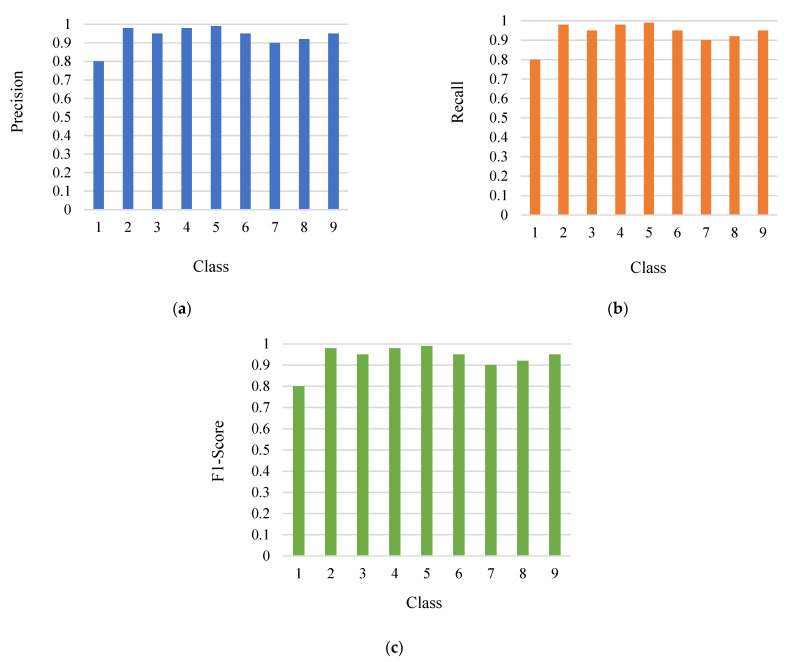
(**a**) Precision; (**b**) Recall; (**c**) F1-Score.

The precision, recall, and F1-Score of the model is shown in Figure 10 (a–c). The performance of parameters is measured by accuracy, but other factors such as precision, recall, and F1-score also contribute to it. These factors are computed for all the classes and shown below in the experiment performed. These factors are calculated on the true positive, true negative, false positive, and false negative values for all the classes. The high precision shows that the accuracy will be increased. A high recall value indicates the number of relevant positive values. The f-score represents the weighted average of the precision and recall.

In Table 3, the proposed approach’s performance is compared to that of three standardized models. The results show that the proposed model outperforms the other classical models using segmentation and an added extra layer in the model.

## 5. Conclusions and Future Scope

The article discussed a deep neural network model for detecting and classifying tomato plant leaf diseases into predefined categories. It also considered morphological traits such as color, texture, and leaf edges of the plant. This article introduced standard profound learning models with variants. This article discussed biotic diseases caused by fungal and bacterial pathogens, specifically blight, blast, and browns of tomato leaves. The proposed model detection rate was 98.49 percent accurate. With the same dataset, the proposed model was compared to VGG and ResNet versions. After analyzing the results, the proposed model outperformed other models. The proposed approach for identifying tomato disease is a ground-breaking notion. In the future, we will expand the model to include certain abiotic diseases due to the deficiency of nutrient values in the crop leaf. Our long-term objective is to increase unique data collection and accumulate a vast amount of data on several diseases of plants. To improve accuracy, we will apply subsequent technology in the future.

## Figures and Tables

**Table 1:** Hyper-parameter of deep neural network.

Parameter	Description
No. of Convolution Layer	8
No. of Max Pulling Layer	8
Dropout Rate	0.5
Network Weight Assigned	Uniform
Activation Function	Relu
Learning Rates	0.01, 0.01, 0.1
Epocho	50, 100, 150
Batch Size	36, 64, 110

**Table 2:** Test results.

Dataset Amount	Image Size	Epoch	Learning Rate	Accuracy (%)
3000	256 × 256 px	50	0.0001	98.47%
		50	0.001	98.42%
		50	0.01	98.52%
		100	0.0001	98.43%
		100	0.001	98.58%
		100	0.01	98.5%

**Table 3:** Comparison with other models.

S.No.	Model	Accuracy Rate	Space	Training Parameters	Non-Trainable
1	Mobinet	66.75	82,566	18,020,552	455,262
2	VGG16	79.52	85,245	21,000,254	532,654
3	InceptionV3	64.25	90,255	22,546,862	658,644
4	Proposed	98.49	22,565	1,422,542	0

## Data Availability

Not applicable.
